# Supplementary material for: Ibrutinib in Combination with Lenalidomide Revlimid/Dexamethasone in Relapsed/Refractory Multiple Myeloma (AFT-15)
Source: Cancers (Basel). 2025 Jul 23;17(15):2433. doi: 10.3390/cancers17152433 (PMC12346632; doi:10.3390/cancers17152433)
Supplement: Supplementary file 1 [file cancers-17-02433-s001.zip › cancers-3680933-supplementary.pdf]

Ibrutinib in Combination with Revlimid/Dexamethasone (Rd) in Relapsed/Refractory Multiple Myeloma (AFT-15)

Supplemental Table S1: Adequate Blood Chemistry

- Absolute neutrophil count (ANC) ≥ 1000/mm^3
- Platelet counts ≥ 75,000/mm^3 (or ≥50,000/mm^3 if bone marrow involvement is ≥50%)
- Hemoglobin ≥ 8 g/dL, independent of transfusion and growth factor support for at least 7 days prior to registration.
- Alanine aminotransferase (ALT) and aspartate aminotransferase (AST) ≤ 2.5 x upper limit of normal (ULN)
- Total bilirubin ≤1.5 x ULN (unless bilirubin rise is due to Gilbert's syndrome or of non-hepatic origin)
- Serum creatinine ≤ 2 x ULN or GFR ≥ 30 ml/min based on either the estimated Glomerular Filtration Rate (Cockcroft Gault) or measured GFR from 24-hour urine sample[4]

Supplemental Table S2: Dose modification for Ibrutinib

**Hematologic Adverse events**	**Action to be Taken**
Grade 4 neutropenia lasting for more than 7 days.<br>Grade 3 thrombocytopenia in the presence of clinically significant bleeding events<br>Grade 4 thrombocytopenia	Hold Ibrutinib until resolved to Grade 1 or baseline and reduce ibrutinib by one dose level.<br><br>If these toxicities persist or recur following two dose reductions, ibrutinib will be discontinued
Grade ≥3 neutropenia with infection/fever.<br>Any other Grade 4 hematological toxicities	Hold Ibrutinib until resolved to Grade 1 or baseline. ibrutinib may be reinitiated. If these toxicities persist or recur following two dose reductions, ibrutinib will be discontinued
**Non-Hematologic Adverse Events**	**Action to be Taken**
Grade 3 or 4 nausea, vomiting, or diarrhea if persistent, despite optimal anti- emetic and/or anti-diarrheal therapy.<br>Any other Grade 4 or unmanageable Grade 3 toxicity.	Hold Ibrutinib until resolved to Grade 1 or baseline and reduce ibrutinib by one dose level.<br><br>If these toxicities persist or recur following two dose reductions, ibrutinib will be discontinued
Any other Grade 3 or 4 non-hematological toxicity	Hold Ibrutinib until resolved to Grade 1 or baseline, ibrutinib may be reinitiated. f these toxicities persist or recur following two dose reductions, ibrutinib will be discontinued
